# Integration of DCE-MRI and DW-MRI Quantitative Parameters for Breast Lesion Classification

**DOI:** 10.1155/2015/237863

**Published:** 2015-08-03

**Authors:** Roberta Fusco, Mario Sansone, Salvatore Filice, Vincenza Granata, Orlando Catalano, Daniela Maria Amato, Maurizio Di Bonito, Massimiliano D'Aiuto, Immacolata Capasso, Massimo Rinaldo, Antonella Petrillo

**Affiliations:** ^1^Department of Diagnostic Imaging, Radiant and Metabolic Therapy, “Istituto Nazionale Tumori Fondazione Giovanni Pascale, IRCCS”, Via Mariano Semmola, 80131 Naples, Italy; ^2^Department of Electrical Engineering and Information Technologies, University “Federico II” of Naples, Via Claudio 21, 80125 Naples, Italy; ^3^Department of Diagnostic and Laboratory Pathology, “Istituto Nazionale Tumori Fondazione Giovanni Pascale, IRCCS”, Via Mariano Semmola, 80131 Naples, Italy; ^4^Department of Senology, “Istituto Nazionale Tumori Fondazione Giovanni Pascale, IRCCS”, Via Mariano Semmola, 80131 Naples, Italy

## Abstract

*Objective*. The purpose of our study was to evaluate the diagnostic value of an imaging protocol combining dynamic contrast-enhanced MRI (DCE-MRI) and diffusion-weighted MRI (DW-MRI) in patients with suspicious breast lesions.* Materials and Methods*. A total of 31 breast lesions (15 malignant and 16 benign proved by histological examination) in 26 female patients were included in this study. For both DCE-MRI and DW-MRI model free and model based parameters were computed pixel by pixel on manually segmented ROIs. Statistical procedures included conventional linear analysis and more advanced techniques for classification of lesions in benign and malignant.* Results*. Our findings indicated no strong correlation between DCE-MRI and DW-MRI parameters. Results of classification analysis show that combining of DCE parameters or DW-MRI parameter, in comparison of single feature, does not yield a dramatic improvement of sensitivity and specificity of the two techniques alone. The best performance was obtained considering a full combination of all features. Moreover, the classification results combining all features are dominated by DCE-MRI features alone.* Conclusion*. The combination of DWI and DCE-MRI does not show a potential to dramatically increase the sensitivity and specificity of breast MRI. DCE-MRI alone gave the same performance as in combination with DW-MRI.

## 1. Introduction

Magnetic resonance imaging (MRI) applications such as dynamic contrast enhanced (DCE) and diffusion weighted imaging (DWI) have the potential to provide noninvasive digital biomarkers with good spatial resolution and reproducibility suitable for early detection of breast cancer and for therapy evaluation [1–9]. In general, DCE-MRI has shown high sensitivity for breast cancer detection (89–100%) [1–12], while DWI has shown utility in predicting suitable therapies and monitoring response [13].

DCE-MRI consists in the serial acquisition of images before and after the injection of intravenous contrast agent; it has been shown to give information about vascular permeability within the tumor [10, 11]. Different methods for DCE-MRI data analysis have been proposed, ranging from simple semiquantitative inspection of the time-intensity curves (TICs) to more sophisticated tracer kinetics modeling [14–18]. The different methods were designed to capture the biologically relevant components from the dynamic MR signal and to relate them to the underlying pathophysiological processes taking place in the tissue. In principle, the derivation of full-quantitative physiological data from DCE-MRI should rely on the application of appropriate tracer kinetics models to describe the distribution of contrast media following its systemic administration. However, the application of these techniques is still complex and they could not be widely available outside specialist centers. In response to this, many semiquantitative approaches for the classification of TIC shapes have been described and are now in relatively common use in clinical settings [18–26].

DW-MRI images are sensitive to water diffusion; preclinical and clinical data showed that it can reflect vessels structure [12, 13]. An approximated quantitative analysis of DW-MRI can be performed calculating the apparent diffusion coefficient (ADC) based on the relative signal intensity change of the tissue with increasing *b* values (see Section 2) or by using intravoxel incoherent motion (IVIM) [27–29] modeling for a more accurate quantitative analysis that has the potential to provide information about both the cellularity and perfusion of tumors. With the increasing awareness of the toxicity of MR contrast agents, DW-MRI could be considered a favorable alternative for deriving perfusion information without contrast agent injection [27–33].

At the time of writing only a few studies explored the correlation between these two methods and attempted a comparison in the case of breast cancer; moreover, it could be useful to assess their independence or complementarity.

The objective of the present study is to evaluate the correlation between DCE-MRI and DW-MRI data in breast cancer; moreover, we tried to establish if opportunely combining DCE and DW-MRI features for differentiation of benign and malignant breast lesions could improve performance.

## 2. Materials and Methods

### 2.1. Patients Characteristics

31 breast lesions (15 malignant and 16 benign, proved by histological examination) in 26 female patients (mean age 37.2 ± 10.4 years, range 14–53 years) were included in this study. The malignant lesions included 9 infiltrating ductal carcinomas, 3 infiltrating ductal-lobular carcinomas, 1 infiltrating lobular carcinoma, and 2 ductal carcinomas in situ (DCIS). The benign lesions included 11 fibroadenomas and 5 fibrocystic dysplasias.

### 2.2. MR Protocol

Per each subject, DW-MRI and DCE-MRI data were acquired consecutively during the same session with a 1.5 T scanner (Magnetom Symphony, Siemens Medical System, Erlangen, Germany) equipped with breast dedicated coil. Scan settings are reported in Table 1.

DW-MRI data comprised 7 scans, each corresponding to a different *b* value (0, 50, 100, 150, 400, 800 and 1000 s/mm^2^).

DCE-MRI data comprised 10 consecutive scans acquired with an interval between two successive scans of 56 s. The contrast agent bolus, 0.1 mL/kg body weight of Gd-DOTA (Dotarem,Guerbet, Roissy CdG Cedex, France), was injected at the start of the first postcontrast scan. An automatic injection system was used (Spectris Solaris EP MR, MEDRAD, Inc., Indianola, PA). The injection flow rate was 2 mL/s followed by a flush of 10 ml saline solution at the same rate.

### 2.3. Volumes Coregistration

A 3D linear interpolation was performed in order to align DCE and DW data on a common grid. Before alignment the voxel size of DCE and DW was 1.44 × 1.44 × 2 mm^3^ and 1.52 × 1.52 × 6 mm^3^, respectively. After the alignment the common spatial resolution was 1.5 × 1.5 × 6 mm. For the subsequent analysis only voxels included in both datasets were considered.

### 2.4. Region of Interest

Region of interests (ROIs) have been manually drawn by an expert radiologist on DCE images with virtual “fat-suppression” obtained subtracting the precontrast from the 5th postcontrast image. Per each patient only the slices including the lesion have been used. Voxels within ROIs were extracted from both DCE and DW realigned volume data. Features from DCE data and DW data have been computed.

### 2.5. DCE-MRI Features

Per each voxel, 20 features were extracted from DCE data: 17 were model free and 3 were model based.

#### 2.5.1. Model Based Features

The time-course of contrast medium concentration is typically modelled using the extended Tofts model [22, 24]:

(1) $$\begin{array}{c} C_{t}\left(\;t,K_{\mathrm{trans}},k_{\text{ep}}\;\right)=C_{p}\left(\;t\;\right)⊗K_{\mathrm{trans}}\cdot e^{-k_{\text{ep}}\cdot t}+v_{p}\cdot C_{p}\left(\;t\;\right), \end{array}$$

where *C*_*t*_(*t*) is the concentration of contrast medium within the tissue (voxel) of interest; *C*_*p*_(*t*) is the concentration of contrast medium within the plasma (also called arterial input function (AIF)); *K*_trans⁡_ is the volume transfer constant from plasma to extracellular-extravascular space (EES); *k*_ep_ is the diffusion rate constant from EES to plasma; *v*_*p*_ is the volume fraction occupied by plasma. These parameters can be related to the level of angiogenic activity. In particular, *K*_trans⁡_ represents the vessel permeability and *k*_ep_ is linked to the duration of the wash-out phase [21]. We assumed the biexponential AIF proposed by Weinmann et al. [34]:

(2) $$\begin{array}{c} C_{p}\left(\;t\;\right)=d\left(\;a_{1}\exp\left(\;-m_{1}t\;\right)+a_{2}\exp\left(\;-m_{2}t\;\right)\;\right), \end{array}$$

where *d* is the administered dose (mL/kg), *a*_1_ = 3.99 kg/L, *a*_2_ = 4.78 kg/L, *m*_1_ = 0.144 min^−1^, and *m*_2_ = 0.0111 min^−1^. Contrast medium concentration was calculated from the TIC using the approach suggested by Schabel et al. [35] with a fixed precontrast longitudinal relaxation time, *T*_1,0_ of 820 ms, appropriate for breast parenchyma.

#### 2.5.2. Model Free Features

For each voxel 17 TIC's shape descriptors were computed using an approach previously reported in [26]: basal signal (SB), maximum signal difference (MSD), the time to peck (TTP), the WI slope (WIS), the WO slope (WOS), the WI intercept (WII), the WO intercept (WOI), the WOS/WIS ratio, and the WOI/WII ratio are under curve (AUC), area under gadolinium curve in the was-in phase (AUCWI), area under gadolinium curve in the wash-out phase(AUCWO), the AUCWO/AUCWI ratio, and the height ratio (HR).

Moreover in this study the perfusion index (PI) as proposed by [36], the sum of intensity difference (SOD) as proposed by [37], and variance of enhancement slope (VES) as proposed by [38] were calculated.

### 2.6. DW-MRI Features

**Figure 1:**
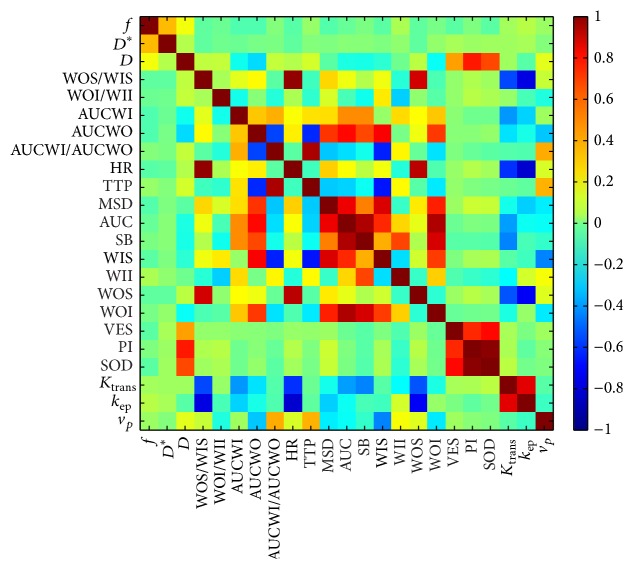
Per each couple of features the Spearman correlation coefficient (*r*) at a voxel-by-voxel level is reported in color code. Yellow to red colors indicate a positive correlation; cyan to blue colors indicate negative correlation. Most of DCE features are not correlated with DW features; a relatively strong positive correlation is observed between *D* and PI (*r* = 0.70) and between *D* and SOD (*r* = 0.60).

Per each voxel, 3 features were extracted from DW data using the Intra Voxel Incoherent Motion (IVIM) model (Figure 1 step 7) [27–29].

DW-MR signal decay is most commonly analyzed using the monoexponential model [26, 27]:

(3) $$\begin{array}{c} \text{ADC}=\frac{\ln\left(S_{0}/S_{b}\right)}{b}, \end{array}$$

where *S*_*b*_ is the MRI signal intensity with diffusion weighting *b*, *S*_0_ is the nondiffusion-weighted signal intensity, and ADC is the apparent diffusion coefficient.

For a voxel with a large vascular fraction, the MRI data decay can deviate from a monoexponential form, in particular showing a fast decay in the range of low *b* values generated by the intravoxel incoherent motion (IVIM) effect [27, 28]. Thus, in addition to the monoexponential model, a biexponential model was used to estimate the IVIM-related parameters of pseudodiffusivity (*D*_*p*_ indicated also with *D* ^∗^), perfusion fraction (*f*_*p*_), and tissue diffusivity (*D*):

(4) $$\begin{array}{c} \frac{S_{0}}{S_{b}}=f_{p}\cdot \exp\left(\;-b\cdot D_{p}\;\right)+\left(\;1-f_{p}\;\right)\cdot \exp\left(\;-b\cdot D\;\right). \end{array}$$

The estimation of the three parameters in the biexponential model may often be ill-conditioned because of a limited number of samples, small perfusion fraction, and/or similar compartmental diffusivities, as found in other in vivo IVIM studies [29–33]. Thus, we performed a “two steps” analysis procedure as follows.

Typically, *D*_*p*_ is greater than *D* [28]; therefore, when the *b* value is significantly greater than ~1/*D*_*p*_ (e.g., for *D*_*p*_ ~ 10 mm^2^/ms, *b* > 100 s/mm^2^), the contribution of the pseudodiffusion term to the signal decay becomes negligible. In this higher *b* value regime, (3) can be simplified to a monoexponential equation (4), where by *D* can be estimated:

(5) $$\begin{array}{c} S_{\text{high}}=S_{0}\cdot \exp\left(\;-b\cdot D\;\right). \end{array}$$

Therefore, *D* is determined from a monoexponential fit to data above a chosen threshold (*b* > 200 s/mm^2^, in this study). After determining *D* using (5), *D*_*p*_, *f*_*p*_ can be estimated using a nonlinear fit of (4) to the entire dataset that minimizes the residual sum of squares.

### 2.7. Statistical Analysis

We performed two types of analysis: voxel-by-voxel and lesion-by-lesion analysis. It is expected that the results of the voxel-by-voxel analysis should quantify the possibility to automatically segment images using a combination of DCE and DW information. The results of the lesion-by-lesion analysis should indicate the capability to discriminate benign from malignant. Therefore, both voxel features and lesion features (per each lesion the median value along all the voxels for all features) have been calculated.  Table 2 summarizes the 23 features.

In order to assess the correlation between DCE and DWI we made an analysis at a voxel-by-voxel level. Specifically, we computed the Spearman (nonparametric) correlation coefficient between each couple of features. It is expected that strongly correlated (or inversely correlated) features should show a high correlation coefficient (approximately 1 or −1).

Subsequently, we used both voxel-by-voxel and lesion-by-lesion analysis in order to assess the capability of the features to discriminate benign from malignant voxels or lesions. We applied a Linear Discriminant Analysis (LDA) [39] followed by a linear classifier in order to identify the best combination of features able to produce best classification results. ROC curves for classification were generated. The best linear classifiers were determined by maximizing the area under the ROC curves; best threshold was identified considering the unbalance of benign-malignant lesions [40, 41].

Finally, the performance of a simple algorithm for lesion classification based on voxel-by-voxel analysis was evaluated: a lesion was classified as malignant (benign) if the majority of voxel within that lesion is classified as malignant (benign). Classification based on DCE alone, DW alone, and combination of DCE and DW was compared. In the three cases, the percentage of correctly classified samples was computed for each lesion.

## 3. Results

Figure 1 shows the Spearman correlation coefficient (*r*) at a voxel-by-voxel level between all feature couples. The numbering of the features is as in Table 2. It can be seen that in general, DCE and IVIM parameters show weak correlation except PI & *D* (*r* = 0.70) and SOD & *D* (*r* = 0.60).

**Figure 2:**
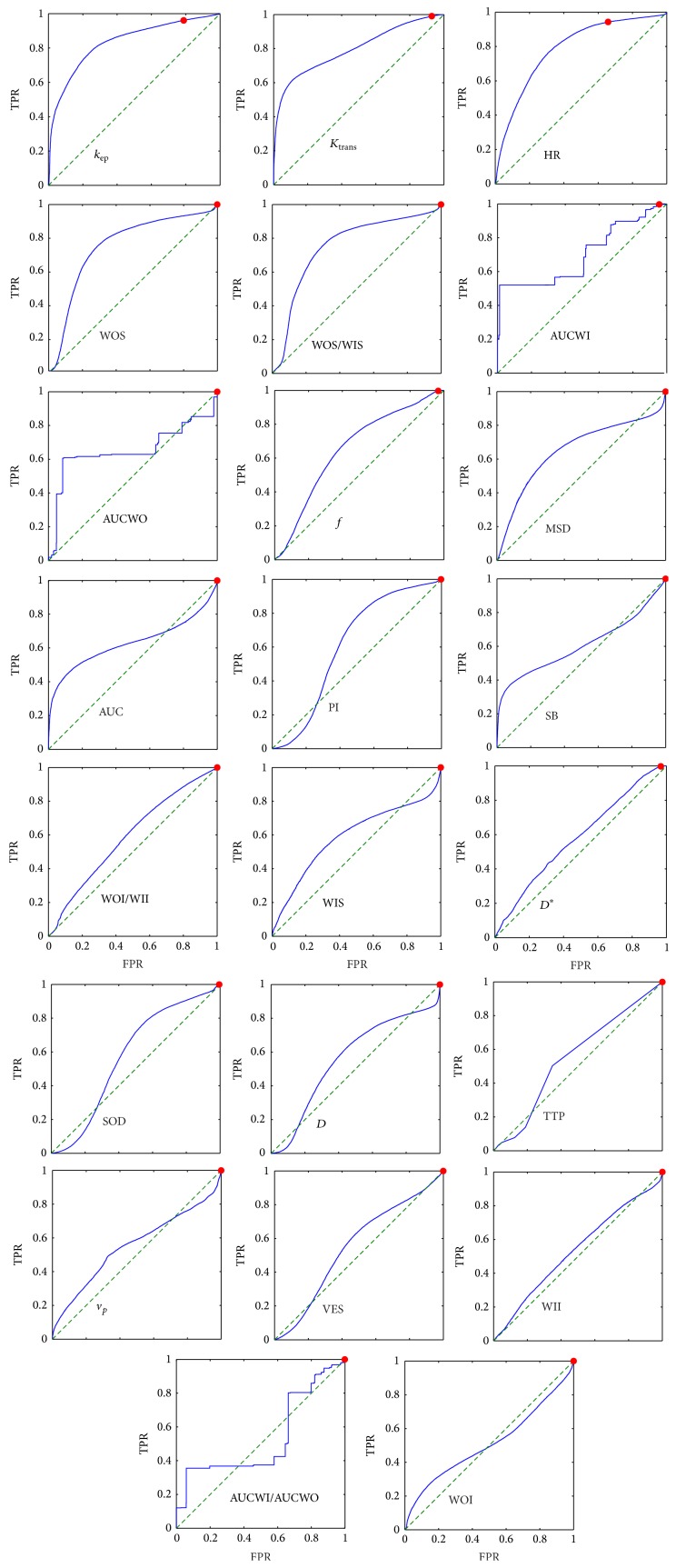
Receiver operating curves (ROCs) of single features in the case of voxel-by-voxel analysis. Per each feature (see Table 2) the ROC is reported in terms of true positive rate (TPR) and false positive rate (FPR). The plots have been aligned according to the area under curve (AUC) in row-wise descending order with the largest AUC at the top-left. The red dot indicates the best compromise between TRP/FPR considering the unbalance between benign-malignant subjects. FPR is generally very high except for *k*_ep_ and HR.

**Figure 3:**
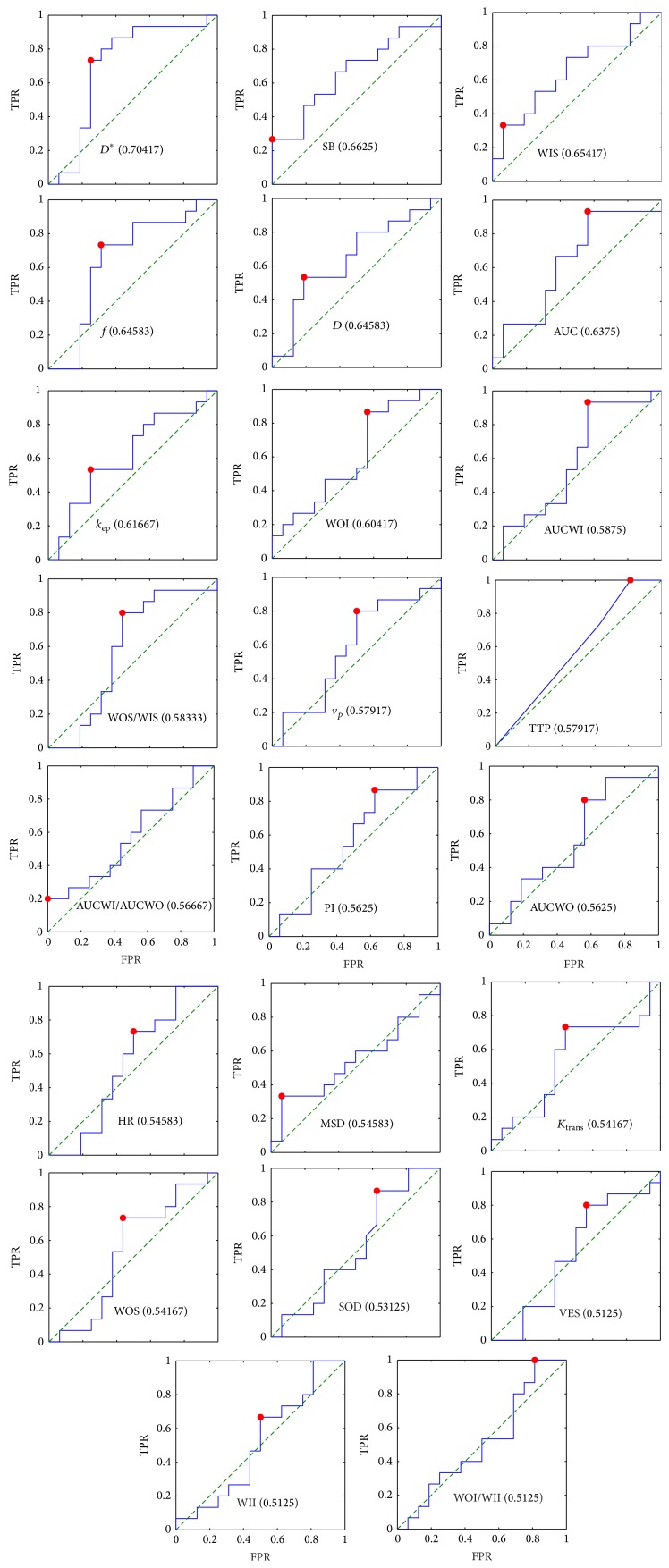
Receiver operating curves (ROCs) of single features in the case of lesion-by-lesion analysis. Per each feature (see Table 2) the ROC is reported in terms of true positive rate (TPR) and false positive rate (FPR). The plots have been aligned according to the area under curve (AUC) in row-wise descending order with the largest AUC at the top-left (per each feature the AUC is indicated in parenthesis). The red dot indicates the best value considering the unbalance between benign-malignant subjects.

Figures 2 and 3 show the receiver operating curves (ROCs) for single features in the case of voxel-by-voxel and lesion-by-lesion analysis, respectively. Within a specific ROC the best threshold is indicated by a red dot which has been evaluated considering the unbalance between benign and malignant lesions. The largest area under curve (AUC) with high sensitivity and good specificity has been obtained for *k*_ep_, *K*_trans⁡_ and HR, in the case of voxel-by-voxel analysis and for *D* ^∗^, WIS, and *f*, in the case of lesion-by-lesion analysis (we discarded SB because of very low sensitivity).

**Figure 4:**
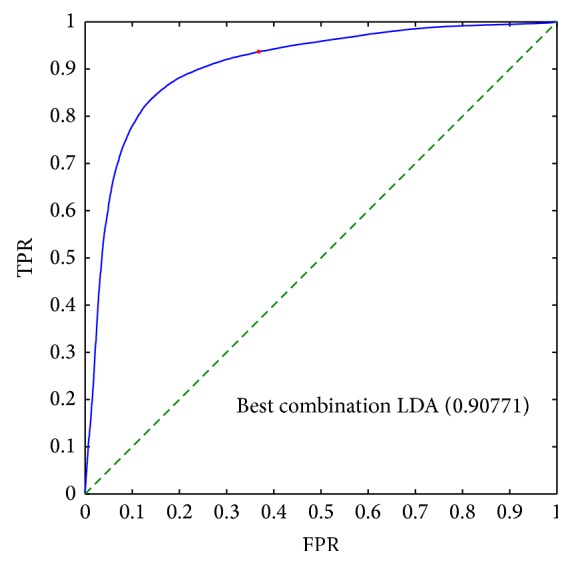
ROC analysis of the best linear combination of* all* features obtained using Linear Discriminant Analysis in the case of pixel-by-pixel analysis. The AUC is indicated in parenthesis. The red dot indicates the best point considering the unbalance between benign-malignant patients: the TPR is approximately 0.93 and the FPR is about 0.35.

**Figure 5:**
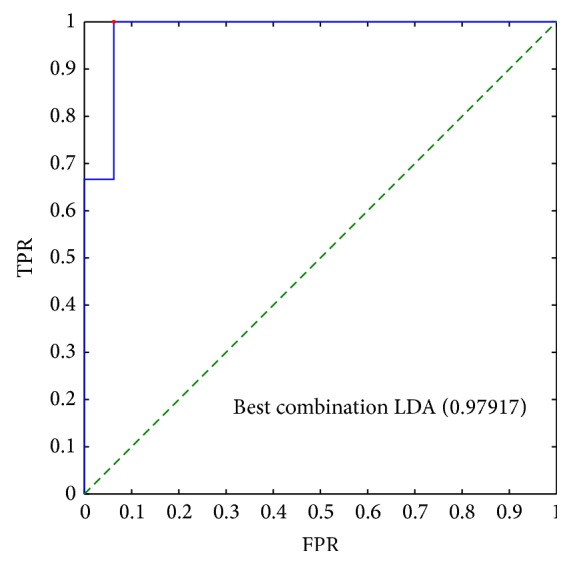
ROC analysis of the best linear combination of* all* features obtained using Linear Discriminant Analysis in the case of lesion-by-lesion analysis. The red dot indicates the best point considering the unbalance between benign-malignant patients. The AUC is reported in parenthesis. The TPR is 1 with FPR less than 0.1.

Figures 4 and 5 report the results of the best combination of all features in the LDA analysis in the case of voxel-by-voxel and lesion-by-lesion analysis respectively. The best threshold is indicated by a red dot, which has been evaluated considering the unbalance between benign and malignant lesions.

**Figure 6:**
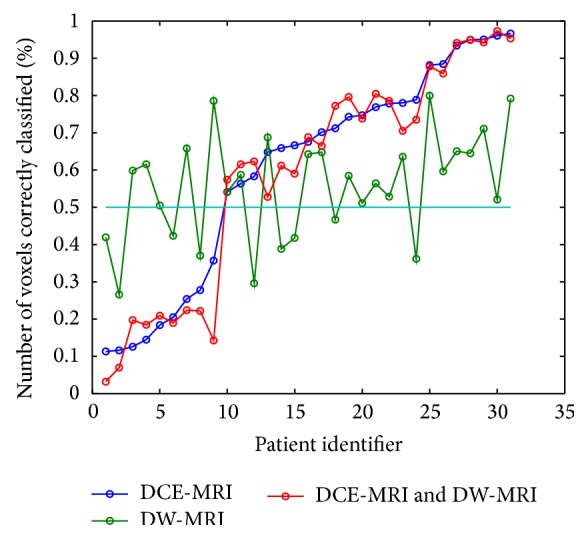
Result of a simple algorithm for classifying benign and malignant lesions: the voxels within a lesion can be classified as benign or malignant using the best combination of features in the pixel-by-pixel analysis: a lesion is classified as malignant if it has a percentage of malignant voxels higher than 50%. Per each patient, the percentage of the correctly classified voxels within the ROI is reported: if this percentage is higher than 50% than the lesion will be correctly identified. It can be seen that using DCE alone (blue line) only 9 lesions have been incorrectly classified. Using only DW we have again 9 lesions misclassified, but they are different from the previous ones. Moreover, the combination of DCE and DW produces the same results as DCE only.

Figure 6 shows the percentage of correctly classified samples by Linear Discriminant Analysis for each lesion. Using DCE features only showed the same behavior of combined DCE & DW. The number of misclassified patients using DCE only and DW only was the same (9 patients were misclassified in both cases).

Tables 3 and 4 report sensitivity and specificity of the parameters in the voxel-by-voxel analysis that provide the maximum area under the ROC (AUROC).

## 4. Discussion

The purpose of our study was to evaluate the diagnostic value of an imaging protocol that combines dynamic contrast-enhanced MRI (DCE-MRI) and diffusion-weighted imaging (DWI) in patients with suspicious breast lesions and to determine if additional information provided by DWI could improve the diagnostic value of breast MRI.

Our findings showed that no strong correlation was obtained between DCE-MRI and DW-MRI features (Figure 1). The largest values were obtained in correspondence of the pairs PI & *D* and SOD & *D*, probably because PI and SOD are the only two features that describe the whole trend of the time intensity curve course, which allow obtaining the best discrimination between the different types of lesion.

The ROC analysis showed that single features (both DCE and DW) do not have a good discriminative power (Figures 2 and 3). Moreover, the unbalance of our data does not allow determining a good threshold with both high sensitivity and specificity.

Results of Linear Discriminant Analysis (Figures 4 and 5) showed that the use of a combination of DCE and DW features, in comparison to single features, has the potential to improve sensitivity and specificity.

However, the potential of both the set of features cannot be achieved using simple classification algorithms such as the one proposed in Section 2.7. Further investigations toward to best way to combine the information from DCE and DW should be performed.

According to our knowledge, no previous study in the literature tried to combine DCE and DW-MRI features including model free and model based parameters evaluated by DCE-MRI data and IVIM parameters evaluated by DW-MRI data, after automatic registration and preprocessing of two volumes, to assess the accuracy in differentiation of benign and malignant breast lesions and to evaluate the improvement of additional of DW-MRI parameters to DCE-MRI features in breast lesion classification.

Several authors in recent literature have combined DCE and DW-MRI data in breast cancer to different aims. Rahbar et al. [42] developed a model incorporating DCE and DW-MRI features, including semimodel free parameters and ADC, to differentiate high-nuclear-grade (HNG) from non-HNG ductal carcinoma in situ (DCIS) in vivo. Those preliminary findings suggested that DCE and DW-MR imaging features may aid in identifying patients with high risk DCIS. Kul et al. [43] evaluated the diagnostic value of an imaging protocol that combined DCE and DW-MRI in patients with suspicious breast lesions and to determine if additional information provided by DWI improves the diagnostic value of breast MRI. They concluded that the combination of DWI and DCE-MRI has the potential to increase the specificity of breast MRI. Partridge et al. [44] showed that ADC can improve the positive predictive value of breast MRI for lesions of varied types and sizes. Jena et al. [45] have tried to evaluate the combined effect of capillary permeability (*K*_trans⁡_) and tissue cellularity (ADC) on the diagnostic accuracy for differentiating benign and malignant breast lesions by incorporating these parameters in routine clinical protocol for breast MRI. Wu et al. [46] reported in their study that the combined use of DW-MRI and CE-MRI has the potential to improve the diagnostic performance in monitoring neoadjuvant chemotherapy (NAC). Atuegwu et al. [47] presented a methodology for incorporating ADC and kinetic DCE-MRI features into a simple mathematical model of tumor growth to predict the tumor cellularity and early treatment response at NAC. In this contribution, results indicate how the integration of DW- and DCE-MRI data can improve specificity and positive predictive value to separate responder by nonresponder patients after one cycle of NAC. It is worth mentioning the recent work proposed by Cai et al. [48, 49]: they proposed a machine learning approach to combining diffusion-weighted imaging (DWI), morphology, and kinetic features from DCE-MRI in order to improve the discrimination power of malignant from benign breast masses. They examined seven features divided in four groups: morphological features, texture features, kinetic features, and one DWI feature (apparent diffusion coefficient). Together with the selected diagnostic features, various classical classification schemes were used to test their discrimination power through cross validation scheme. They concluded that multisided variables, which characterize the morphological, kinetic, pathological properties, and DWI measurement of ADC, could improve the discriminatory power of breast lesions.

However, some drawbacks must be underlined: in particular, they used a nonlinear classifier combining the seven features with support vector machine, Bayesian classifier, *k*-nearest neighbours, and logistic regression model, all approaches that determine a nonlinear manipulation of features; this latter is not easy to understand by radiologists with respect to a linear combination of features. Moreover, in our study, in contrast to Cai et al. [48, 49], we combined features including model free and model based parameters evaluated by DCE-MRI data and DW-MRI data.

A few remarks must be made: in our study model based parameters were used because they are more strongly related to physiological characteristics of the tissue (perfusion). We did not considered morphological features, as done in Cai et al. [48, 49], because we were interested only in functional aspects of the lesion: this could explain differences in results between our study and cited studies that included also morphological and textural features.

A major limitation of our study is the small size of the population: an increase is required to increase the power of the statistical tests and to detect statistical differences between the two groups (benign and malignant lesions).

Although not conclusive, our results seem to suggest that the combined use of DCE-MRI and DW-MRI does not provide a dramatic improvement compared to the use of DCE-MRI features alone, in the classification of breast lesions.

## Figures and Tables

**Table 1:** Scan settings.

Settings	DCE-MRI	DW-MRI	Units
TR/TE/*α*	9, 8/4, 76/25	7700/129/90	ms/ms/deg
Pulse sequence	T1-weighted 3D FLASH	T2-weighted SPAIR	—
Plane	Coronal	Axial	—
FOV	185 × 370	183 × 360	mm^2^
Matrix size	128 × 256	120 × 236	pixel
Pixel spacing	1.44 × 1.44	1.52 × 1.52	mm^2^
Slice thickness	2	4	mm
Gap between slices	0	2	mm
Number of slices	80	24	—

**Table 2:** Summary of DCE and DW features.

ID	Symbol	Description	Units
1	*f*	Perfusion fraction	—
2	*D* ^∗^	Pseudodiffusion coefficient	mm^2^ s^−1^
3	*D*	Tissue diffusion coefficient	mm^2^ s^−1^
4	WOS/WIS	Ratio between slopes of wash-out and wash-in phase (see below)	—
5	WOI/WII	Ratio between intercepts of wash-out and wash-in phase (see below)	—
6	AUCWI	Area under gadolinium curve in the wash-in phase	s mmol L^−1^
7	AUCWO	Area under gadolinium curve in the wash-out phase	s mmol L^−1^
8	AUCWI/AUCWO	Ratio between areas of wash-out and wash-in phase	—
9	HR	Height ratio	—
10	TTP	Time to peak	s
11	MSD	Maximum signal difference	—
12	AUC	Area under curve	—
13	SB	Basal signal	—
14	WIS	Wash-in slope	s^−1^
15	WII	Wash-in intercept	—
16	WOS	Wash-out slope	s^−1^
17	WOI	Wash-out intercept	—
18	VES	Variance of enhancement slope	s^−1^
19	PI	Perfusion Index	—
20	SOD	Sum of intensity difference	—
21	*K*_trans⁡_	Volume transfer constant from plasma to extracellular-extravascular space	s^−1^
22	*k*_ep_	Diffusion rate constant from extracellular-extravascular space to plasma	s^−1^
23	*v*_*p*_	Plasma volume fraction	—

**Table 3:** Sensitivity and specificity of the parameters in the pixel-by-pixel analysis that provide the maximum area under the ROC (AUROC).

Parameters	AUROC	Sensitivity	Specificity
*k*_ep_	0.7	0.96	0.22
*K*_trans⁡_	0.66	0.99	0.18
HR	0.65	0.94	0.35

**Table 4:** Sensitivity and specificity of the parameters in the lesion-by-lesion analysis that provide the maximum area under the ROC (AUROC). We have discarded SB because of low sensitivity.

Parameters	AUROC	Sensitivity	Specificity
*D* ^∗^	0.7	0.73	0.75
WIS	0.65	0.33	0.94
*f*	0.64	0.73	0.69
